# Acute exercise exacerbates ischemia-induced diastolic rigor in hypertensive myocardium

**DOI:** 10.1186/2193-1801-1-46

**Published:** 2012-11-02

**Authors:** Patricia O Reger, Stephen C Kolwicz, Joseph R Libonati

**Affiliations:** 1 Department of Biokinetics, Eastern University, St. David’s, Radnor Township, PA USA; 2 Mitochondria and Metabolism Center, School of Medicine, University of Washington Seattle, Seattle, WA USA; 3 Biobehavioral and Health Sciences, School of Nursing, University of Pennsylvania, 135 Claire M. Fagin Hall, 418 Curie Boulevard, Philadelphia, PA 19104-4217 USA

**Keywords:** Exercise, Preconditioning, Hypertension, Heart, Ischemia

## Abstract

Previous studies have shown that acute exercise preconditions the myocardium from ischemic injury. The purpose of this study was to test whether acute exercise protects the hypertensive myocardium from ischemia-induced diastolic rigor, and to compare the response between normotensive and uncompensated hypertensive hearts. Hearts harvested from female Wistar-Kyoto (WKY; n = 24) and spontaneously hypertensive rats (SHR; n = 27) (age:10–12 weeks) were exposed to ischemia (Langendorff isovolumic preparation; 22 minutes of no flow ischemia and studied following prior conditions of: 1) no exercise (WKY-CON, n=8; SHR-CON, n=8); 2) ischemia initiated one hour post-acute exercise (WKY-1HR, n = 8; SHR-1HR, n = 11); and 3) ischemia initiated 24 hours post-acute exercise (WKY-24HR; n = 8; SHR-24HR, n = 8). Acute exercise consisted of one bout of treadmill running at 25 m/min for 60 minutes. Heart weight was similar between WKY and SHR despite elevated *in vivo* resting systolic blood pressure and rate pressure product in SHR (P<0.05). During normoxic perfusion, left ventricular (LV) Langendorff performance was similar between WKY and SHR over the post-exercise time course. However, during ischemia, LV diastolic rigor was less in WKY vs. SHR (P<0.05). Acute exercise augmented ischemia-induced LV dysfunction one hour post-exercise in SHR (P<0.05), with gradual recovery by 24 hours post-exercise. These data suggest that acute exercise promotes ischemic diastolic rigor in SHR, even prior to the development of cardiac hypertrophy.

## Introduction

There are numerous cardiovascular health benefits associated with regular participation in exercise and physical activity. Growing attention, however, has been placed on how single acute exercise sessions influence cardiovascular function. For example, strenuous exercise in humans has been shown to promote cardiac fatigue (Oxbourough et al. 2010; Starnes and Bowles 1995; Scharag et al. 2008) and tissue damage in the heart (George et al. 2004) with several studies showing transient cardiac functional decrements (Dawson et al. 2007) and increased plasma concentrations of cardiac-specific troponins following exercise (George et al. 2004; Urhausen et al. 2004; Trivax et al. 2010). While factors such as the duration/intensity of exercise (Urhausen et al. 2004), training status (Neilan et al. 2006), gender (Scott et al. 2006; Scott & Warburton 2008), and environmental factors (Shave et al. 2004) are all significantly involved in the magnitude of the reported post-exercise dysfunction, the underlying cellular events remain elusive. One hypothesis is that acute aerobic exercise promotes oxidative stress and apoptosis in the heart (Huang et al. 2009; La Gerche et al. 2007) and this contributes to post-exercise cardiac dysfunction.

Conversely, many studies have shown that acute aerobic exercise protects the heart from subsequent metabolic insults. For example, acute exercise has been reported to precondition the heart from subsequent ischemia-reperfusion injury by attenuating experimentally-induced infarct size (Brown et al. 2005; Domenech et al. 2002; Taylor et al. 1999; Yamashita et al. 2001). Exercise induced cardioprotection has been observed in both rat (Brown et al. 2005; Yamashita et al. 2001) and dog myocardium (Yamashita et al. 2002) and is temporally associated with both early and delayed ischemic preconditioning, that might be dependent upon the generation of heat shock proteins. Thus the literature suggests that acute aerobic exercise elicits various reponses, with cardioprotection juxtaposed to temporal periods of cardiac dysfunction/damage.

We sought to examine some of these important issues in the present paper. For our experiments, we tested the potential deleterious or cardioprotective influences of acute exercise on ischemic cardiac function in normotensive and hypertensive hearts. We studied hypertensive hearts because the increased workload associated with chronic hypertension has a profound effect on myocardial metabolism and may influence post-exercise ischemic tolerance. Given the importance of understanding the impact of acute exercise on intrinsic cardiac function, we performed our studies in Langendorff-isolated hearts. In our studies, we specifically examined; i) the temporal effects of acute exercise on intrinsic cardiac function under normoxic conditions, ii) the temporal effects of acute exercise on ischemic left ventricular diastolic performance, and iii) the temporal effects of acute exercise in the hypertensive myocardium under normoxic and ischemia conditions. We hypothesized that a single bout of exercise would have limited effects in altering cardiac function under normoxic conditions, but would protect both normotensive and hypertensive myocardium from the development of ischemic-induced diastolic rigor.

## Materials and methods

### Animals and acute exercise protocol

Female Wistar-Kyoto (WKY; n = 24) and Spontaneously Hypertensive Rats (SHR; n = 27) (age:10–12 weeks) rats were obtained from Charles River Laboratories (Germantown, NY). All rats were housed 3 per cage, maintained on a 12-h light/dark cycle, and fed ad libitum (Harlan Teklad Global Diets, 18% Protein Diet, Madison, WI.) The animals were randomly studied under the following conditions: 1) no exercise (WKY-CON, n=8; SHR-CON, n=8); 2) one hour post-acute exercise (WKY-1HR, n = 8; SHR-1HR, n = 11); and 3) 24 hours post-acute exercise (WKY-24 HR; n = 8; SHR-24 HR, n = 8). The acute exercise protocol consisted of the animals running at low to moderate intensity on a motorized rodent treadmill at 25 m/min at 0% grade for 60 minutes. Animals in the 1 HR group were killed one hour after exercise. Animals in the 24 HR group were killed 24 hours after exercise. All animals were maintained in accord with institutional standards and in accord with the “Principles of Laboratory Animal Care” formulated by the National Society for Medical Research and the “Guide or the Care and Use of Laboratory Animals” prepared by the Institute of Laboratory Animal Resources and published by the National Institutes of Health.

### *In vivo* heart rate and blood pressure measurements

*In vivo* heart rates (HR) (mean of 25 cardiac cycles) and systolic blood pressures (SBP) were collected prior to exercise and within two minutes after the completion of the acute bout of exercise in a subset of animals, utilizing standard tail cuff techniques previously described (MacDonnell et al. 2005).

### Langendorff isolated heart preparation

Rats were anesthetized with sodium pentobarbital (50 mg/kg; IP) and heparinized intravenously (500 U; IV). The heart was excised, trimmed of excess tissue, and rapidly immersed in cold (4°C), Ca ^2+^-free Krebs-Henseleit buffer (KHB). Hearts were placed on a Langendorff perfusion apparatus (ML785B2, ADInstruments, Colorado Springs, CO) and perfused at 16 ml/min (STH pump controller ML175, ADInstruments, Colorado Springs, CO) with a modified Krebs-Henseleit solution containing in mM; 2.0 CaCl_2_, 130 NaCl, 5.4 KCl, 11 dextrose, 2 pyruvate, 0.5 MgCl_2_, 0.5 NaH_2_PO_4_, 25 NaHCO_3_. The buffer was equilibrated with 95% O_2_ and 5% CO_2_ which maintained the pH at 7.35-7.4 as previously described (MacDonnell et al. 2005; Reger et al. 2006).

The coronary flow rate was selected to mimic the in situ perfusion pressure. After coronary perfusion was initiated, the left ventricle (LV) was immediately decompressed with an apical puncture via the insertion of a short apical drain. A balloon was inserted into the LV and the LV balloon volume was adjusted to approximately 11 mmHg of LV end-diastolic pressure (LVEDP) for stabilization. Following stabilization no further alterations in balloon volume were made and baseline LV performance was recorded. Timed measurements of LV pressure (LVP), the maximum rate of positive and negative change in LV pressure (± dP/dt), and coronary perfusion pressures (CP) were continuously made via a data acquisition system (Powerlab/8SP, ADInstruments, Colorado Springs, CO). Coronary perfusion pressure was measured at heart level via a fluid filled pressure transducer. LVDevP was calculated by subtracting the LV end-diastolic pressure (LVEDP) from the LV systolic pressure. To assess LV diastolic performance during ischemia, coronary flow was stopped via a stopcock to produce no flow ischemia. Ischemia persisted for 22 minutes and timed measurements of LV pressures, the maximum rate of positive and negative change in LV pressure (± dP/dt), and coronary perfusion pressures were continuously made.

### Tissue water content measurement

In a subset of experiments, we sought to determine whether acute exercise induced cardiac edema. Thus we determined myocardial tissue water content in a subset of animals (WKYCON, N=3; WKY-1HR, N=3; SHR-CON, N=3; SHR-1HR, N=3). After one hour of recovery from exercise, rats were anesthetized with sodium pentobarbital (50 mg/kg; IP) and heparinized intravenously (500 U; IV). The heart was excised, trimmed of excess tissue and rinsed in cold (4°C), Ca ^2+^-free Krebs-Henseleit buffer (KHB) and weighed. The heart was then passively desiccated at 37.5°C until a stable dry weight was achieved. Tissue water content was calculated as ([wet weight-dry weight]/dry weight) and expressed as ml H_2_O/gm dry weight as previously described by our group (Mohara et al. 2005).

### Data analysis

Animal characteristics at the time of sacrifice were compared with student t-tests. ANOVA followed by Tukey post hoc analyses were used to analyze LV performance at baseline and during ischemia, respectively. All analyses were performed using SPSS version 12.0 (Chicago, IL). Significance was set at an alpha level of *P* < 0.05. Data are reported as the mean ± SE.

## Results

### *In vivo* hemodynamics

**Figure 1:**
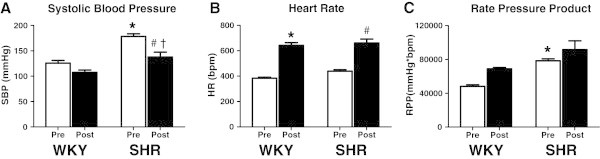
**Blood pressure, heart rate and rate pressure product.** Open bars represent pre-exercise conditions. Closed bars represent post-exercise conditions. Wistar Kyoto (WKY), SHR Spontaneously Hypertensive (SHR). Data are mean ± SE. * P < 0.05 vs. WKY-Pre, # P < 0.05 vs. SHR–Pre, † P < 0.05 vs. WKY–Post.

Systolic blood pressure (SBP), heart rate (HR), and rate pressure product (RPP) in response to acute treadmill running are illustrated in Figure 1. At rest SBP was significantly greater (P<0.05) in SHR compared to WKY. After 60 minutes of exercise, SBP in SHR remained significantly greater compared to WKY (P<0.05), despite relative post-exercise hypotension occurring in both groups. Resting HR tended to be elevated in SHR, with a significant increase noted in the HR response to exercise in both groups (P<0.05). The RPP at rest was significantly increased in SHR relative to WKY at rest, and tended to be higher in SHR following exercise.

### Animal characteristics

The physical characteristics of the groups are presented in Table 1. Prior to death, the body weight (BW) was significantly less in SHR relative to WKY (P<0.0001). Heart weight (HW), HW/BW ratio, tibial length (TL) and HW/TL were similar between groups. Post exercise myocardial H_2_O content was also comparable between groups (Table 2).

**Table 1:** **Animal characteristics**

	WKY	SHR
	N = 24	N = 27
BW (g)	174 ± 2.2	159 ± 1.6*
HW(mg)	941 ± 39	914 ± 22
HW/BW (mg/g)	5.4 ± 0.2	5.7 ± 0.1
TL (mm)	31.6 ± 0.3	31.0 ± 0.2
HW/TL (mg/mm)	27.4 ± 1.1	29.4 ± 0.7

Data are presented as mean ± SE. Abbreviations; *WKY* Wistar Kyoto; *SHR* spontaneously hypertensive rat ; *N* no. of animals; *BW* Body Weight; *HW* Heart Weight; *TL* Tibial Length. * Significantly different from WKY, P<0.0001.

**Table 2:** **Myocardial water content**

	***WKY***-***CON***	***WKY***-***1HR***	***SHR***-***CON***	***SHR***-***1HR***
	***N*** = ***3***	***N*** = ***3***	***N*** = ***3***	***N*** = ***3***
**Water Content** (**ml**/**gram dry wt**)	3.1 ± 0.01	3.1 ± 0.08	3.2 ± 0.06	3.1 ± 0.04

Data are presented as mean ± SE. Abbreviations; *WKY* Wistar Kyoto; *SHR* SHR; *CON* Control; *1HR* hearts harvested one hour after exercise.

### Langendorff isolated heart performance

#### Normoxia

**Figure 2:**
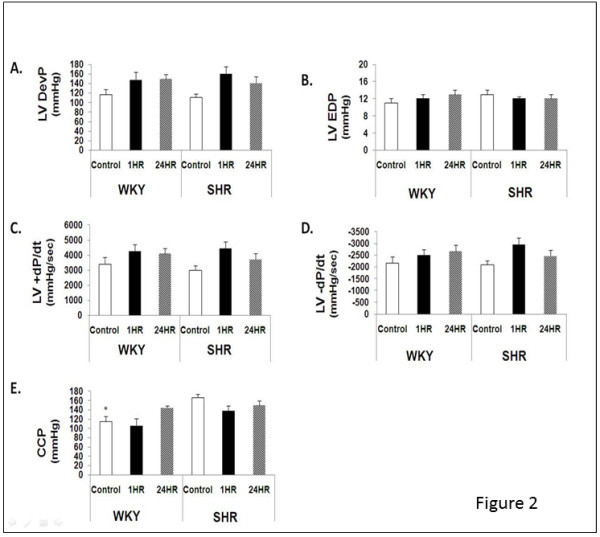
**Post-exercise Langendorff performance during normoxia.** Data are presented as mean ± SE. Abbreviations; WKY, Wistar Kyoto; SHR, SHR; CON, Control; 24HR, hearts24 hours after exercise; 1HR, hearts one hour after exercise; LVDevP, left ventricular developed pressure; LVEDP, left ventricular end diastolic pressure; LV+dP/dt, rate of force development; LV-dP/dt, rate of relaxation; CP, coronary perfusion pressure. * Significantly different from SHR-CON, P<0.05.

Figure 2 illustrates the normoxic Langendorff, isolated heart performance in the WKY and SHR groups following exercise. Cardiac function (LV DevP, LVEDP, and ± dP/dt) was not statistically different between groups. In our model, all hearts were perfused at a constant coronary flow rate of 16 ml/min with a crystalloid perfusate. This constant flow allows for differences in perfusion pressure to be illustrative of coronary vascular resistance. In control hearts at baseline, coronary perfusion pressure (CP) was higher in SHR-CON relative to WKY-CON indicative of increased coronary vascular resistance (Figure 2E, P< 0.05). This observation remained in the immediate period post-exercise as CP was significantly higher in SHR-1HR vs. WKY-1HR (Figure 2E). Interestingly, CP was similar in WKY and SHR 24 hours post exercise (Figure 2E).

#### Ischemia

**Figure 3:**
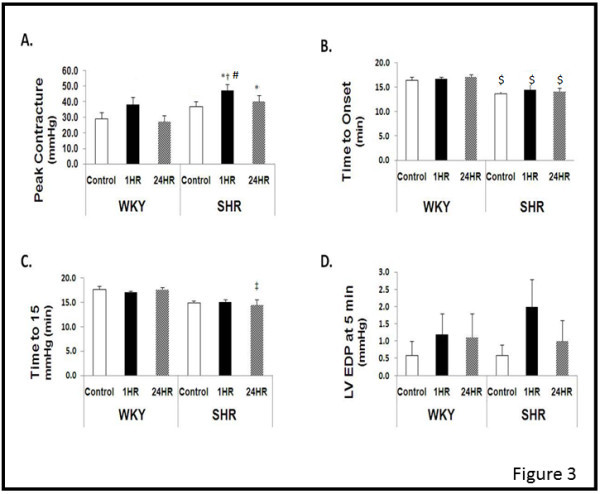
**Post -exercise Langendorff performance during ischemia.** Data are presented as mean ± SE. Abbreviations; WKY, Wistar Kyoto; SHR, SHR; CON, Control; 24HR, animals sacrificed 24 hours after exercise; 1HR, animals sacrificed one hour after exercise;. *Significantly different from WKY-CON, P<0.05. † Significantly different from WKY-1HR, P<0.05. ‡ Significantly different from WKY-24, P<0.05. # Significantly different from SHR-CON, P<0.05. $ Significantly different from WKY-Control, WKY-1HR, and WKY24 HR, P<0.05.

Figure 3 illustrates the LV diastolic response during no flow ischemia. Relative to WKY-CON, peak contracture was significantly greater in SHR-1HR and SHR-24HR (P<0.05), with a tendency (P=0.10) to be greater in WKY-1HR and SHR-CON (Figure 3A). Additionally, the time of onset to ischemic contracture occurred significantly earlier in SHR vs. WKY in control, 1HR, and 24HR hearts (Figure 3B). Likewise, the ischemic time required to reach 15 mmHg occurred earlier in SHR-24HR vs. WKY-24 HR (Figure 3C,P<0.05). However, during the early course of ischemia (i.e., LVEDP at 5 min), there were no statistical differences among groups (Figure 3D).

## Discussion

Our findings suggest that acute exercise does not induce intrinsic cardiac dysfunction in Langendorff isolated hearts during normoxic perfusion. However, when coronary flow was compromised during global ischemia, left ventricular (LV) diastolic performance was impaired in SHR following exercise. This is evidenced by increasing the magnitude of rigor development one hour post exercise. The time to onset of rigor was also reduced in SHR at all study intervals relative to WKY. These data suggest that low to moderate levels of acute aerobic exercise do not impair intrinsic cardiac function when coronary blood flow is well maintained, but potentiates diastolic dysfunction during ischemia in hypertensive hearts, even without evidence of compensatory hypertrophy.

Hypertension is a well-established risk factor for the development of coronary artery disease, ischemia, and heart failure. Sustained elevations in arterial blood pressure result in the development of left ventricular hypertrophy and apoptosis (Kolwicz et al. 2009), and have been associated with an increased myocardial vulnerability to metabolic stress (Taegtmeyer and Overturf 1988). It is well documented that ischemia induces functional impairments in cardiomyocyte relaxation. This impairment, known as ischemic contracture, occurs as the actin-myosin cross-bridges fail to dissociate, their attachments persist, and tension is therefore maintained throughout diastole resulting in increased diastolic stiffness (Jennings and Reimer 1981, Libonati et al. 1997). The contracture that develops during ischemia is, in part, thought to result from low ATP concentrations. When ischemia persists, further declines in intracellular ATP [ATP]_i_ precipitates a rise in intracellular calcium ([Ca2+]_i_) which in turn leads to additional rigor and signals for cell damage (Allen and Orchard 1983, Allen and Smith 1985, and Cobbold and Bourne 1984). In our studies, hypertensive hearts showed increased myocardial workloads, i.e. rate pressure product with exercise, which may be associated with increased [ATP]_i_ turnover and may underlie the worsened ischemic diastolic dysfunction.

The increased workload associated with chronic hypertension has a profound effect on myocardial metabolism. It has been reported the myocardial PCr/ATP ratio determined at rest is below normal when LV mass is increased (Neubauer et al. 1997; Zhang et al. 1993; Zhang et al. 1995). In fact, even in the absence of LVH, overloaded hearts demonstrated abnormalities in high energy phosphate metabolism (Lortet et al. 1993). It has been suggested that these alterations in high energy phosphate metabolism are due to decreases in creatine kinase (CK) activity (Smith et al. 1990). Moreover, an intact CK system has been identified as being critical in the maintenance of calcium homeostasis and LV function under metabolically stressed conditions (Spindler et al. 2004). Chronic hypertension also impacts the fuel selection of the heart as decreased fatty acid oxidation and increased rates of glycolysis have been observed in the hypertrophied myocardium (Kolwicz et al. 2012, Raizda et al. 1993; Yonekura et al. 1985). Similar findings have been reported in experiments from non-hypertrophied, hypertensive hearts in which a preferential use of glucose in comparison to fats, increases in glycolytic enzymes, and decreases in ketone metabolic enzymes have been noted (Taegtmeyer and Overturf 1988).

Myocardial ischemic preconditioning has been shown to profoundly protect the heart from post-ischemic myocardial dysfunction and infarction during a subsequent ischemic episode (Yellon and Downey 2003). Similarly, some studies, although limited in number, have shown that exercise can protect the heart against ischemia-reperfusion injury in a matter similar to that of ischemic preconditioning (Brown et al. 2005; Domenech et al. 2002; Taylor et al. 1999; Yamashita et al. 2001). These studies demonstrated that exercise results in both early and delayed exercise-induced preconditioning as evidenced by a reduction in myocardial infarct size in rats (Brown et al. 2005, Yamashita et al. 2001), and dogs (Domenech et al. 2002) as well as enhanced myocardial performance 24 hours after exercise (Taylor et al. 1999). Conversely, (Locke et al. 1995) failed to observe improved post-ischemic LV contractile performance 24 hours after a single bout of exercise, while (Huang et al. 2009) showed that exhaustive endurance training impaired LV function and promoted apoptosis in rats.

In our study, a single bout of exercise was not only ineffective in protecting the myocardium from rigor development during ischemia, but it also reduced LV tolerance to ischemia one hour after exercise in SHR. The compromised ischemic tolerance after exercise and the lack of exercise-induced protection is difficult to explain, as it is in conflict with the majority of the aforementioned ischemic preconditioning studies and is contrary to our initial hypothesis. It should be noted that the term “preconditioning” generally refers to hearts are that are exposed to ischemia and reperfusion with the subsequent assessment of myocardial damage. In the current study we only examined diastolic performance during prolonged ischemia without reperfusion. Thus differences in experimental paradigms and animal models are important to appreciate in comparing our results to the existent literature. It should also be noted that the exercise modality (treadmill running), intensity (15–30 meters/min in previous reports), and duration (up to 60 minutes in previous reports) was similar between the present study and prior reports (Brown et al. 2005, Yamashita et al. 2001, Taylor et al. 1999, Locke et al. 1995).

Even though the young SHR hearts in our study did not show compensated hypertrophy, other intrinsic metabolic and humeral factors may have predisposed these hearts to post-exercise diastolic rigor during ischemia. For example, SHR animals have high levels of sympathetic tone (Kuo et al. 2012), which can prompt an elevated myocardial oxygen consumption during and following acute exercise relative to WKY. Thus underlying metabolic differences with pressure overload may be unveiled with a metabolic stress like post exercise-induced ischemia. However, one interesting result of this study is the finding that acute exercise reduced the systolic blood pressure in SHR rats. This effect was not related to the ex vivo cardiac function which was modified neither by the strain nor by acute exercise in pre-ischemic conditions. A reduction in peripheral resistance was likely involved in the relative hypotension in SHR immediately post exercise. In the present study we determined whether acute exercise, caused myocardial edema. Our results showed that neither WKY nor SHR tissue water content was increased immediately post exercise; thereby edema does not seem to underlie the increased ischemic diastolic rigor in SHR. More work is needed to establish the underlying mechanisms.

Strenuous exercise in humans has been shown to promote cardiac fatigue (Oxbourough et al. 2010; Starnes and Bowles 1995; Scharag et al. 2008) and tissue damage in the heart (George et al. 2004, Urhausen et al. 2004; Trivax et al. 2010). While increasing attention has been placed on how single acute exercise sessions influence cardiovascular function, little work has been done in hypertensive hearts. Our findings are significant in that while factors such as the duration/intensity of exercise (Urhausen et al. 2004), training status (Neilan et al. 2006), gender (Scott et al. 2006: Scott & Warburton 2008), and environmental factors (Shave et al. 2004) are all involved in the magnitude of post-exercise cardiac dysfunction, pathological heart phenotypes, i.e. hypertension, also need to be considered.

The SHR model was chosen for our study because it closely mimics the clinical course of untreated hypertension in humans. It has been documented that concentric hypertrophy happens in SHR between 4 and 12 months of age, decompensating to heart failure at approximately 15 months (Boluyt et al. 1994). We chose to study these animals at 10 weeks of age because hypertension is established yet no significant compensatory hypertrophy is present. Thus our results suggest that factors unrelated to cardiac hypertrophy underlie the increased post exercise diastolic impairments during ischemia in hypertension. The limitations to the SHR model are two-fold; 1) the causes of hypertension in SHR are polygenic and do not necessarily reflect the genetic anomalies associated with hypertension in humans and 2) we did not account for the hormonal influence of estrogen. Despite these limitations, the present data suggest that there appears to be a temporal component to the physiological stress of exercise, which includes a period of increased susceptibility to myocardial ischemic injury, and that this increased vulnerability is greater in the young female SHR uncompensated heart. We propose that this response may be metabolic in nature, but more work is needed to identify the underlying mechanisms.
